# A multi-omics based anti-inflammatory immune signature characterizes long COVID-19 syndrome

**DOI:** 10.1016/j.isci.2022.105717

**Published:** 2022-12-05

**Authors:** Johannes J. Kovarik, Andrea Bileck, Gerhard Hagn, Samuel M. Meier-Menches, Tobias Frey, Anna Kaempf, Marlene Hollenstein, Tarik Shoumariyeh, Lukas Skos, Birgit Reiter, Marlene C. Gerner, Andreas Spannbauer, Ena Hasimbegovic, Doreen Schmidl, Gerhard Garhöfer, Mariann Gyöngyösi, Klaus G. Schmetterer, Christopher Gerner

**Affiliations:** 1 Department of Internal Medicine III, Clinical Division of Nephrology and Dialysis, Medical University of Vienna, Waehringer Gürtel 18-20, Vienna 1090, Austria; 2 Joint Metabolome Facility, Faculty of Chemistry, University of Vienna, Waehringer Straße 38, 1090 Vienna, Austria; 3 Department of Analytical Chemistry, Faculty of Chemistry, University of Vienna, Waehringer Straße 38, 1090 Vienna, Austria; 4 Department of Laboratory Medicine, Medical University of Vienna, Waehringer Gürtel 18-20, Vienna, Austria; 5 Division of Biomedical Science, University of Applied Sciences FH Campus Wien, Vienna, Austria; 6 Department of Medicine II, Division of Cardiology, Medical University of Vienna, Waehringer Gürtel 18-20, Vienna, Austria; 7 Department of Clinical Pharmacology, Medical University of Vienna, Vienna, Austria

**Keywords:** Immunology, immune response, omics

## Abstract

To investigate long COVID-19 syndrome (LCS) pathophysiology, we performed an exploratory study with blood plasma derived from three groups: 1) healthy vaccinated individuals without SARS-CoV-2 exposure; 2) asymptomatic recovered patients at least three months after SARS-CoV-2 infection and; 3) symptomatic patients at least 3 months after SARS-CoV-2 infection with chronic fatigue syndrome or similar symptoms, here designated as patients with long COVID-19 syndrome (LCS). Multiplex cytokine profiling indicated slightly elevated pro-inflammatory cytokine levels in recovered individuals in contrast to patients with LCS. Plasma proteomics demonstrated low levels of acute phase proteins and macrophage-derived secreted proteins in LCS. High levels of anti-inflammatory oxylipins including omega-3 fatty acids in LCS were detected by eicosadomics, whereas targeted metabolic profiling indicated high levels of anti-inflammatory osmolytes taurine and hypaphorine, but low amino acid and triglyceride levels and deregulated acylcarnitines. A model considering alternatively polarized macrophages as a major contributor to these molecular alterations is presented.

## Highlights

• Patients with LCS show a consistent pattern of anti-inflammatory molecules in blood plasma
• This proteomics and metabolomics pattern may serve for diagnostics and monitoring
• Alternatively polarized macrophages may account for responsible pathomechanisms

## Graphical abstract

**Figure:**
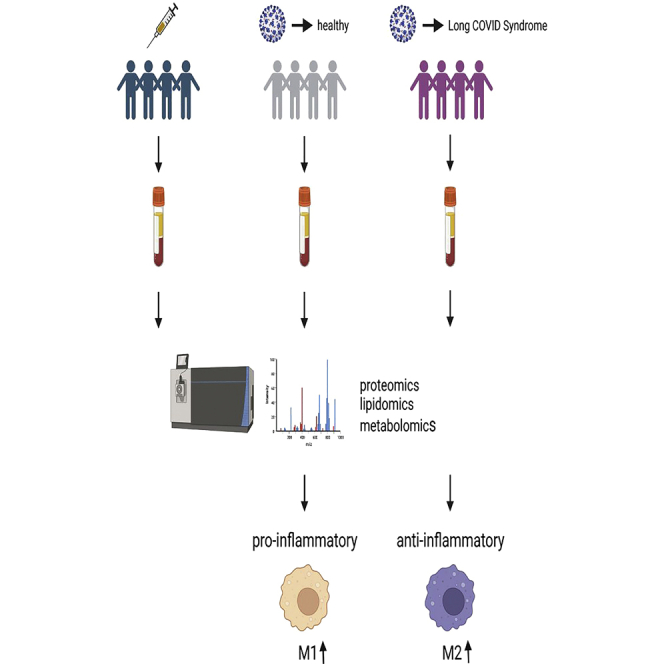

## Introduction

The outbreak of the COVID-19 pandemic in late 2019 has led to an unprecedented worldwide health crisis with the rapid spread of a novel pathogenic member of the coronavirus family (termed SARS-CoV-2) infecting more than 500 million people worldwide. Acute SARS-CoV-2 infection may induce an inappropriate and unique inflammatory response causing pathognomonic severe respiratory symptoms which can be further accompanied by damage to multiple organs such as brain, heart, and kidneys.^1^^,^^2^^,^^3^ Accordingly, acute COVID-19 infection may cause high lethality claiming more than 6 million deaths worldwide so far (www.who.int/emergencies/diseases/). Since the start of the pandemic, it has also become evident, that not all patients fully recover following SARS-CoV-2 infection. At first, the observed symptoms were mainly attributed to psychological conditions such as anxiety and stress in the affected individuals.^4^ However, it is now recognized that chronic persistence of COVID-19 symptoms after acute infection constitutes a novel somatic disease entity termed post-acute COVID-19 syndrome (PACS) or long COVID-19 syndrome (LCS).^5^ Typically, patients with LCS suffer from general fatigue, lack of concentration (self-described as “brain fog”) and physical fitness, dyspnea, postural tachycardia as well as a broad range of other clinical symptoms throughout the whole organism, which severely impedes the quality of life.^6^^,^^7^ These clinical presentations mirror the situations found in chronic fatigue syndrome (CFS) which can be secondary to infection with different viruses and was also during previous coronavirus epidemics including Middle East Respiratory Syndrome (MERS) and Severe Acute Respiratory Syndrome (SARS). Especially for the latter symptom persistence for up to two years has been observed.^8^ Strikingly, the development of LCS is not associated with disease severity and so far, potential risk factors and associated comorbidities are poorly understood.^6^^,^^9^^,^^10^ Similarly, the pathogenesis and pathophysiology of this disease remain rather elusive to date. The limited set of available studies on LCS have indicated tissue damage accompanied by chronic inflammation following recovery from acute COVID-19 infection exacerbation of pre-existing (auto)immune-pathologies or persistence of SARS-CoV-2 at distinct sites in the body.^11^^,^^12^ Yet, neither general consent about the predisposition leading to LCS nor consent about the optimal therapeutic management of the disease has emerged so far. This may also be owed to the rather heterogeneous presentation of symptoms and the lack of awareness of this condition, which allows the speculation that most cases so far remain unrecognized. In light of the high infection rate worldwide, it can be expected that the prevalence of LCS will massively increase in the future, leading to a further COVID-19-associated long-term challenge and burden for the healthcare system. Accordingly, a definition of biomarkers for the diagnosis of LCS as well as in-depth studies for the characterization of pathophysiological processes are clearly warranted. Thus, we here set up an exploratory study to perform broad scale mass spectrometry-based multi-omics analysis of blood specimens from healthy donors after vaccination (termed healthy; H), patients with COVID-19 who had fully recovered (termed recovered; R) and compared them to plasma samples of patients with LCS characterized by lack of concentration, fatigue and associated symptoms (termed long COVID-19; LC) The combination of proteomics and metabolomics focusing on oxylipin analyses has already been demonstrated to strongly support the investigation of pathomechanisms in various diseases.^13^^,^^14^^,^^15^ Using this versatile analysis approach, we were able to identify anti-inflammatory and hypo-metabolic signatures in the proteome, lipidome, and metabolome of LCS patients, thereby providing insights into the molecular regulation and pathophysiology of LCS.

## Results

### Patient characteristics and study design

The first study group consisting of 13 healthy individuals with no history of SARS-CoV-2 infection was recruited three months after full anti-SARS-CoV-2 immunization which was also confirmed by anti-N (−)/anti-S (+) status (termed healthy; H). The second recruited group consisted of 13 age- and gender-matched individuals who had a SARS-CoV-2 infection history at least three months prior to inclusion into this study, but were symptom free in anamnesis at the time of blood draw (termed recovered; R). Infection status was confirmed by positive anti-N and anti-S antibody levels. The third group of 13 study patients had similarly succumbed to PCR-positive SARS-CoV-2 infection at least three months before presentation. All patients in this group had a positive anamnesis of chronic fatigue and/or severe chronic lack of concentrations following SARS-CoV-2 infection combined with at least one more chronic symptom including dyspnea, coughing, and loss of smell among others at the time of presentation, qualifying them for the diagnosis LCS (whole patient characteristics are displayed in Tables 1A and 1B). Again, post-infection status was confirmed by positive antibody testing for anti-N and anti-S antibodies. For the here described exploratory study, specimens from 13 individuals from each group were selected for multi-omics analysis including a cytokine array, untargeted shotgun proteomics, an untargeted eicosanoid/docosanoid analysis, and a targeted metabolomics assay. Routine laboratory testing of basic protein, lipid, and lipoprotein parameters showed no differences between the three groups. Similarly, CRP levels in all three groups were below or only minimally above the threshold (Table 2). Furthermore, none of the analyzed individuals presented with fever or displayed any clinical signs of systemic infection at the time of blood withdrawal, ruling out major systemic metabolic or acute inflammatory processes in the tested individuals at this time point.

**Table 1:** Study group characteristics

Table 1A	healthy (n = 13)	recovered (n = 13)	long COVID (n = 13)
General data			
Gender (f:m)	7:6	7:6	9:4
Age [years]	30 (25–43)	32 (24–49)	33 (21–53)
Time after exposure [months]	6 (3–8)	10 (3–12)	7 (3–10)

Chronic disease (total number (percentage))			

Asthma bronchiale	0	1 (0.07)	4 (0.31)
Multiple sclerosis	0	0	1 (0.07)
Autoimmune thyreoiditis	0	1 (0.07)	1 (0.07)
Atopic dermatitis	0	1 (0.07)	0
Psoriasis arthritis	1 (0.07)	0	0

Table 1B	healthy (n=13)	recovered (n=13)	long COVID (n=13)
**Chronic Symptom (total number (percentage))**			

Lack of concentration	–	–	10 (0.77)
Fatigue	–	–	9 (0.69)
Dyspnoea	–	–	9 (0.69)
Chronic cough	–	–	2 (0.15)
Muscular aching	–	–	3 (0.23)
Amnesic aphasia	–	–	5 (0.38)
Sleep disorder	–	–	4 (0.31)
Urinary incontinence	–	–	2 (0.15)
Nausea	–	–	3 (0.23)
Tinnitus	–	–	4 (0.30)
Other symptoms	–	–	9 (0.69)

**Table 2:** Routine serum parameters (mean values; ranges)

	healthy (n = 13)	recovered (n = 13)	long COVID (n = 13)
total protein [g/L]	72.7 [65.2–77.5]	73.10 [67.5–78.7]	75.3 [63.5–84.3]
albumin [g/L]	49.7 [46.3–54.4]	48.5 [41–53.4]	50.7 [43.7–55.6]
triglyceride [mg/dL]	58 [36-166]	115 [49-353]	77 [48-159]
cholesterol [mg/dL]	171 [108-198]	207 [146-281]	176 [100-192]
HDL [mg/dL]	60 [36-94]	61 [49-93]	54 [37-74]
LDL [mg/dL]	85.2 [53.6–129.2]	102.6 [76.2–201.2]	98.2 [38.6–122.8]
LP(a) [nmol/L]	18 [0-235]	13 [0-421]	15 [0-135]
CRP [mg/L]	0.4 [0–5.7]	1 [0-3]	0.6 [0–6.2]
ferritin [μg/L]	51 [15.1–175.1]	91 [22.4–565]	52.6 [18.7–207.6]

To confirm our hypothesis regarding the role of fatty acids in LCS (see later in discussion) and to exclude an effect from oral supplementation, we analyzed samples from the fourth group including 10 healthy subjects who had not been exposed to SARS-CoV-2. For this purpose, these subjects received tablets containing 870 mg Omega-3 (including 420 mg EPA and 330 mg DHA) twice daily in a prospective study design for one week and plasma samples were obtained before the start of intake and after intake of the last dose.

### Immune activation marker profiling displays a lack of systemic inflammation in patients with long COVID-19 syndrome

**Figure 1:**
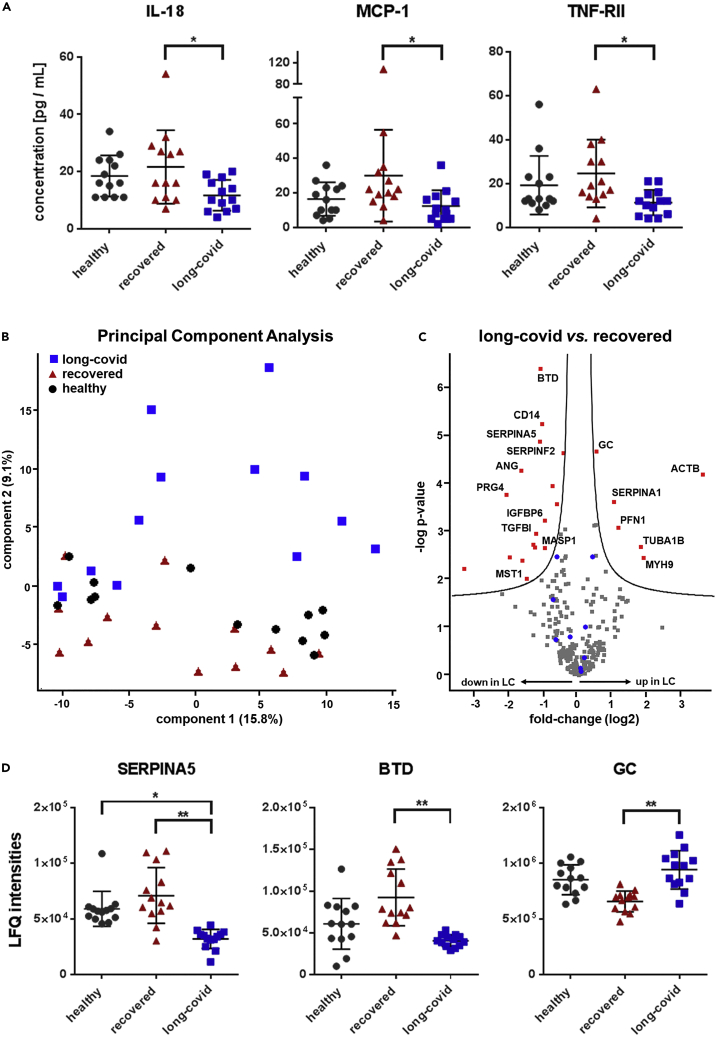
Cytokine and proteome profiles demonstrate a lack of pro-inflammatory activities in patients with long COVID-19 (A) Significant differences of IL-18 as well as the macrophage-derived cytokines MCP-1 and TNF-RII between long COVID-19 and fully recovered patients are depicted. (B) A Principal Component Analysis based on proteome profiling of plasma samples from patients with long COVID-19(blue square), fully recovered patients (red triangle) and vaccinated healthy controls (gray circle) is shown. (C) Significant differences in plasma protein abundance between long COVID-19 and fully recovered patients are visualized by a volcano plot. (D) Label-free quantification (LFQ) intensities derived from untargeted plasma proteomics of SERPINA5, Biotinidase (BTD) and Vitamin D-binding protein (GC) are depicted for all study groups. ∗p ≤ 0.05, ∗∗p ≤ 0.01 according to one-way ANOVA tests.

In order to investigate whether LCS may result from still unresolved inflammatory processes after the viral infection, a panel of 65 cytokines, chemokines, and soluble receptors associated with immune activation was assayed. Remarkably, neither pro- nor anti-inflammatory cytokines were found significantly up-regulated in patients with LCS compared to the other two groups. However, remarkable was the down-regulation of IL-18 in patients with LCS, as this T cell and macrophage-derived pro-inflammatory cytokine is orchestrating migration and antiviral response of macrophages (Figure 1A) Furthermore, the monocyte/macrophage-derived factors MCP-1/CCL-2 and sTNF-RII, were found significantly down-regulated in patients with LCS compared to fully recovered patients (Figure 1A). Overall, cytokine levels were rather low in patients with LCS, indicating a lack of pro-inflammatory activities.

### Proteome profiling displays an anti-inflammatory pattern in patients with long COVID-19 syndrome

To further elucidate the inflammatory status in patients with LCS, we performed shotgun proteomics of plasma samples of the three study groups following an established protocol.^16^ Principal component analysis (PCA) distributed the proteome profiles of healthy vaccinated individuals mainly between the almost completely separated groups of LCS and recovered patients (Figures 1B, S1 and Table S1). Thus, significant (FDR <0.05) proteome alterations were mainly observed comparing the latter two groups (Figure 1C). Apart from several cell leakage products typically derived from uncontrolled cell death, including actin (ACT), tubulin (TUBA1B), myosin-9 (MYH9), and profilin (PFN1), only alpha-1-anti trypsin (SERPINA1) and vitamin D binding protein (GC; Figure 1D) were found significantly up-regulated in the LCS group. A larger number of proteins was found down-regulated, including the protease inhibitors SERPINA5 and SERPINF2, Biotinidase (BTD; Figure 1D), and the macrophage-associated proteins soluble CD14, ANG, and Proteoglycan 4 (PRG4). The acute phase protein CRP was only detected (near threshold levels) in a few samples and thus removed upon filtering. Other acute phase proteins including serum amyloid A and serum amyloid P component, fibrinogens, orosomucoid, and alpha-2-macroglobulin were readily detectable but slightly down-regulated in patients with LCS. These findings further demonstrate the absence of systemic inflammation in patients with LCS and potentially indicate shifts in the activation of inflammatory immune subsets.

### Fatty acid and oxylipin analysis indicated increased phospholipase A2 activities

**Figure 2:**
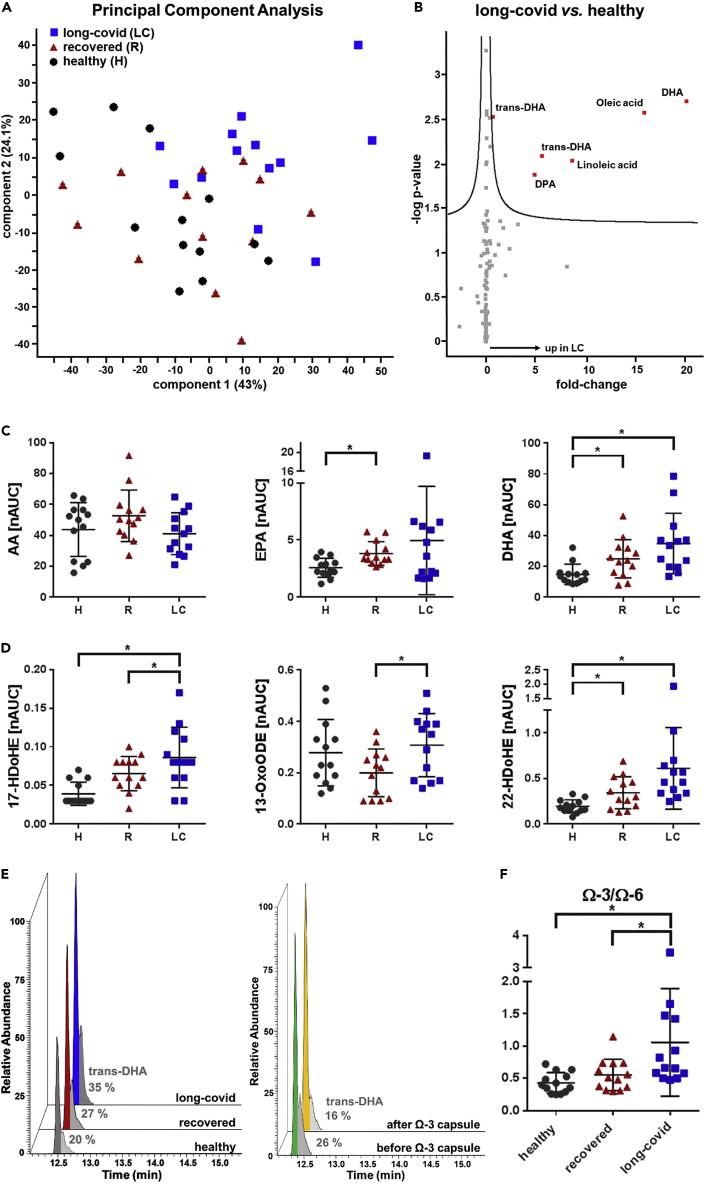
Increased levels of fatty acids with a prevalence for Ω3-fatty acids and anti-inflammatory docosanoids is characteristic for long COVID-19 (A) A Principal Component Analysis based on eicosanoid analysis of plasma samples from patients with long COVID-19 (blue square), fully recovered patients (red triangle) and vaccinated healthy controls (gray circle) is shown. (B) Significant differences in plasma eicosanoids between patients with long COVID-19 and vaccinated healthy controls are visualized by a volcano plot. (C and D) Normalized area under the curve (nAUC) values of arachidonic acid (AA), eicosapentaenoic acid (EPA), docosahexaenoic acid (DHA) and the eicosanoids 17-HDoHE, 13-OxoODE as well as 22-HDoHE are depicted for each study group (H, vaccinated healthy controls; R, fully recovered patients; LC, patients with long COVID-19). (E) Increased levels of *trans*-DHA in patients with long COVID-19 are shown in an extracted ion chromatogram (*m*/*z* = 327.2330 at a retention time (Time) of 12.5 min). No increase in *trans*-DHA levels after nutritional supplementation of omega-3 (Ω-3) capsules was observed in healthy controls. Percentage (%) of *trans*-DHA to DHA is depicted in gray for each study group. (F) A significant increase in the omega-3 to omega-6 ratio (Ω-3/Ω-6) was observed in patients with long COVID-19 compared to vaccinated healthy controls as well as fully recovered patients. ∗p ≤ 0.05 according to two-sided *t*-tests.

Oxidized products of polyunsaturated fatty acids such as arachidonic acid (AA) represent highly bioactive but short-lived signaling molecules and lipid mediators, often termed eicosanoids. Phospholipase A2 catalyzes the first step of biosynthesis, the release of polyunsaturated fatty acids from cell membrane phospholipids. The bioactive oxylipins are subsequently formed by the action of cyclooxygenases, lipoxygenases, or cytochrome P450 monooxygenases and modulate numerous physiological functions including bronchial and vascular tonus, thrombocyte function, inflammation, and other immune responses.^17^ Thus, the assessment of the lipidome allows insights into diverse physiological and pathophysiological processes. Following the analysis of plasma eicosanoids from the three study groups, PCA of fatty acids and their oxidation products separated healthy controls from patients with LCS, with the group of recovered patients dispersed in between (Figure 2A and Table S2). Thus, here most of the significant events were observed comparing patients with LCS to healthy controls (Figure 2B). Evidently, generally higher levels of all kinds of polyunsaturated fatty acids were characteristic of LCS, pointing to higher phospholipase A2 activities. However, increased plasma levels of the pro-inflammatory AA were mainly observed in the recovered group (Figure 2C). In contrast, LCS was marked by the predominant release of the anti-inflammatory molecules EPA and DHA into the blood of affected patients (Figures 2B and 2C). Plasma levels of these molecules may derive from intracellular sources but can also be affected by confounders such as nutrition. Two independent observations indicate that this was not the case in the studied group of patients with LCS. First, the DHA oxidation products 17- and 22-HDoHE were also found significantly increased in patients with LCS (Figure 2D). Second, polyunsaturated fatty acids may occur together with their *trans*-isoforms, which typically stem from nitric oxide signaling.^18^ In an independent group of 10 healthy volunteers, we observed that the nutritional supplementation of DHA was not associated with increased levels of *trans*-DHA (Figure 2E). In contrast, the levels of *trans*-DHA were consistently increased in patients with LCS, suggesting that DHA was preferentially released from intracellular sources. Accordingly, the overall ratio of Ω3-fatty acids to Ω6-fatty acids was found significantly increased in patients with LCS (Figure 2F). A general anti-inflammatory lipid mediator pattern in patients with LCS was also corroborated by increased levels of the 15-LOX product from linoleic acid, 13-OxoODE,^19^ when compared to recovered patients (Figure 2E). Thus, here we provide evidence that increased plasma levels of anti-inflammatory oxylipins may be a characteristic feature of patients with LCS.

### Metabolomic aberrations relate to chronic fatigue syndrome and display an osmolyte-mediated anti-inflammatory signature

**Figure 3:**
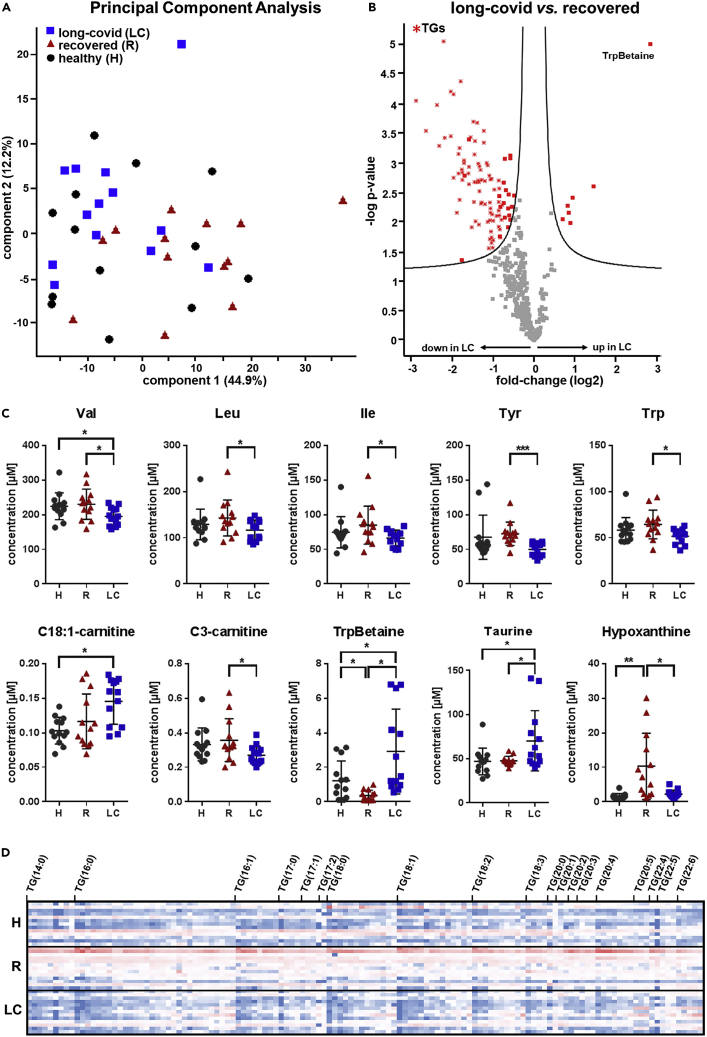
Plasma metabolomics reveals disturbances in the energy metabolism of patients with long COVID-19 (A) A Principal Component Analysis based on metabolomic analysis of plasma samples from patients with long COVID-19 (blue square), fully recovered patients (red triangle) and vaccinated healthy controls (gray circle) is shown. (B) Significant higher levels of TrpBetaine as well as significant lower levels of triacylglycerols (TGs, red star) in plasma samples of patients with long COVID-19 are visualized by a volcano plot. (C) Plasma levels of the amino acids tyrosine (Tyr), tryptophan (Trp), valine (Val), leucine (Leu) and isoleucine (Ile) as well as of carnitines, hypoxanthine, TrpBetaine and taurine are depicted for each study group (H, vaccinated healthy controls; R, fully recovered patients; LC, patients with long COVID-19). (D) A heatmap of all identified triacylglycerols (TGs) displays higher levels of TGs in fully recovered patients compared to patients with long COVID-19 and vaccinated healthy controls. ∗p ≤ 0.05, ∗∗p ≤ 0.01, ∗∗∗p ≤ 0.001 according to two-sided *t*-tests.

In case of metabolites, PCA analysis indicated maximal contrast between recovered and patients with LCS (Figure 3A) similar to the plasma protein analyses described above. Indeed, out of 474 metabolites and lipids analyzed, 107 were found significantly regulated between these two groups (Figure 3B and Table S3). Most apparently, a large number of metabolic alterations were related to a disturbance in energy metabolism, as evidenced by the down-regulation in the LCS group of branched chain amino acids Val, Leu, and Ile, the aromatic amino acid Tyr and amino acids Trp and Arg which also act as inflammation mediators (Figures 3C and S2). Furthermore, increased levels of C18:1-acylcarnitine accompanied by a down-regulation of C3-acylcarnitine were observed in patients with LCS (Figure 3C). This pattern may indicate reduced levels of beta-oxidation accompanied by increased amino acid catabolism. A further indication of disturbed energy metabolism in patients with LCS was the significant increase in lactate, pointing to increased anaerobic glycolysis (Figure S2 and Table S3). Furthermore, the majority of lipids including various triacylglycerols, glycosylceramides, glycerophospholipids, and several ceramides were found down-regulated in LCS (Figure 3D and Table S4), which bears resemblance to a pattern characteristic for chronic fatigue syndrome (CFS).^20^ Remarkably, an LCS-associated lack of inflammatory processes was also evidenced by the present metabolomics results. The metabolite most significantly up-regulated in patients with LCS was hypaphorine (TrpBetaine), an anti-inflammatory alkaloid^21^ described to induce sleep in mice.^22^ Hypaphorine may act as osmolyte similar to the anti-inflammatory molecule taurine (Figure 3C), which was also significantly up-regulated in LCS. In contrast, hypoxanthine levels were strongly increased in the recovered group while patients with LCS showed comparable levels to the healthy vaccinated group.

#### Alternatively polarized macrophage-like cells display features of the long COVID-19 syndrome-associated anti-inflammatory signature

**Figure 4:**
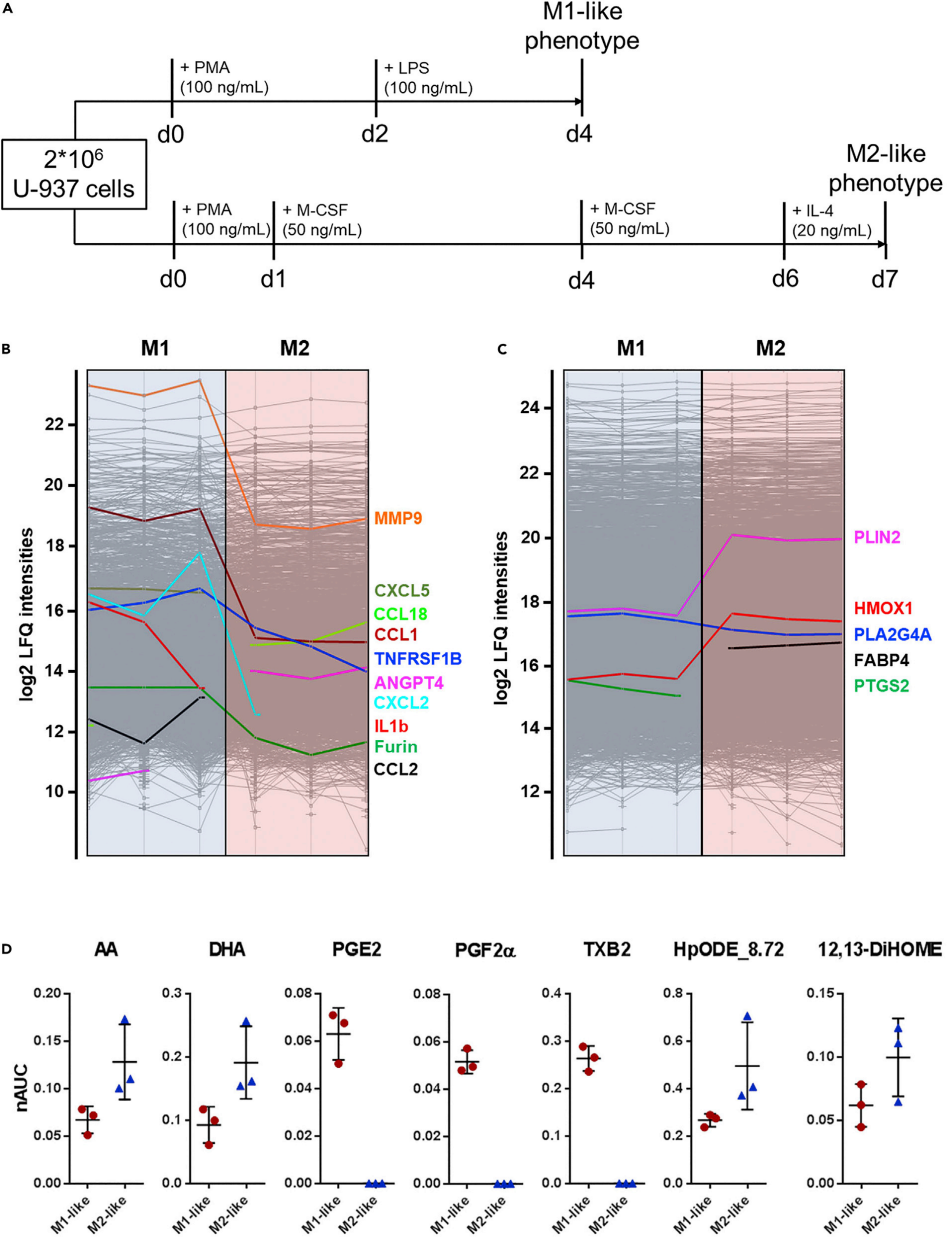
Multi-omics of *in vitro* polarized macrophages reveal molecular features similar to the LCS (A–C) (A) Scheme of *in vitro* polarization of U-937 cells to either M1-like or M2-like macrophages (d, days). Log2-transformed label-free quantification (LFQ) intensities derived from untargeted proteomics of (B) the secretome and (C) the cell lysates of M1-like macrophages (M1) and M2-like macrophages (M2) are depicted using profile plots of the three replicates per condition. (D) Normalized area under the curve (nAUC) values of arachidonic acid (AA), docosahexaenoic acid (DHA) and the eicosanoids PGE2, PGF2a, TXB2, HpODE as well as 12,13-DiHOME in the secretome of *in vitro* polarized M1-like and M2-like macrophages are shown.

A systemic inhibition of inflammatory processes as presently observed in the LCS group may result from a predominance of alternatively polarized macrophages.^23^ To address this issue, we investigated a U937 cell-based macrophage *in vitro* model system again applying proteome and eicosanoid/docosanoid analysis. U937 cells were differentiated into macrophages using PMA and polarized into either M1-like cells by LPS or M2-like cells with GMCF and IL-4 (Figure 4A). As expected, the M1-like cells were found to robustly produce pro-inflammatory cytokines such as IL-1beta, CCL1, CXCL2, CXCL5, and other inflammation marker such as MMP9 (Figure 4B). Indeed, two of the cytokines down-regulated in LCS, MCP-1 (CCL2) and TNFRSF1B (see Figure 1A), were found mainly secreted by M1-like cells and rather down-regulated in M2-like cells (Figure 4B). M1-like macrophages also secreted the protease furin (Figure 4B). Furin was described to cleave the SARS-CoV-2 spike protein in order to allow SARS-CoV-2 virus particles to enter human cells. The main furin inhibitor, SERPINA5, was found significantly down-regulated in patients with LCS (Figure 1D), pointing to a relevant pathomechanism. The M2-like cells displayed a tolerogenic phenotype illustrated by the expression of the immune suppressor CCL18^24^ and the angiogenesis-promoting factor ANGPT4. In addition, the M2-like cells expressed several proteins promoting lipolysis (PLA2G4A), altered lipid metabolism (PLIN2 and FABP4), and lipid peroxidation (HMOX1) (Figure 4C). HMOX1 is actually both an essential enzyme for iron-dependent lipid peroxidation during ferroptotic cell death^25^ and an antiviral protein.^26^ Polarized macrophages also released fatty acids and their oxidation products. Both arachidonic acid and DHA were released by M2-like cells more than by M1-like cells (Figure 4D). In line with the specific expression of COX2 (PTGS2) in M1-like cells, the COX-products PGE2, PGF2alpha and TXB2 were only detected in M1-like cells (Figure 4D). On the other hand, the lipid peroxidation products HpODE and the cytochrome P450 product 12,13-DiHOME were found at higher levels released from M2-like cells. Thus, the molecular patterns observed in patients with LCS are effectively mimicked by M2 macrophages *in vitro*.

## Discussion

Post-acute sequelae of SARS-CoV-2 infection (termed long COVID-19 syndrome) can be found in about 10% of affected patients and thus pose an ever-increasing burden. So far, the pathophysiology of LCS is unknown and subject to speculative hypotheses such as persisting chronic inflammation after infection.^12^ Given this lack of information we chose to perform a broad-scale exploratory study assessing the proteome, lipidome and metabolome in patients with LCS. As control groups, we recruited individuals who had fully recovered after acute COVID-19 infection and healthy individuals after COVID-19 vaccination. Blood plasma of these groups were obtained around three months after vaccination or PCR confirmed SARS-CoV-2 infection to specifically assess the resolution of inflammation. These analyses allowed us to identify LCS specific molecular patterns which strongly point at several unexpected processes of LCS pathophysiology.

Numerous studies have clearly established that acute COVID-19 infection is associated with hyperinflammation. Thus, a failure to clear such inflammatory activities has been commonly assumed to account for LCS symptoms.^12^ A recent study employing multi-omics and single cell transcriptomics with regard to LCS suggests several risk factors and a specific role of T cells in patients with gastrointestinal complications.^11^ Here, we focused on patients with LCS reporting chronic fatigue syndrome or similar symptoms. We present evidence suggesting systemic anti-inflammatory conditions in patients with LCS after the acute infection with SARS-CoV-2. A lack of pro-inflammatory activities and the predominance of anti-inflammatory mediators in blood plasma was independently confirmed at the levels of cytokines, acute phase proteins, oxylipins and metabolites. Furthermore, metabolomics analyses indicated a sustained catabolic metabolism in patients with LCS, which may account for the characteristic chronic fatigue symptoms.

Of the detected cytokines, chemokines and soluble receptors, the three markers IL-18, soluble TNF-RII and MCP-1/CCL2 were significantly down-regulated in the LCS group. All three factors have pro-inflammatory functions and reflect the activation and communication of T-lymphocytes and monocytes/macrophages. In the proteome, down-regulation of acute phase proteins was observed, which was most pronounced between the recovered and the LCS group. Of note, SERPINA5 levels were significantly decreased in the LCS group compared to both the healthy as well as the recovered group. SERPINA5 serves as antagonist of the protease furin, which is essential for viral entry into human cells.^27^ Thus, it is intriguing to speculate that SERPINA5 may represent a predictive biomarker indicating an increased risk for the development of LCS.

**Figure 5:**
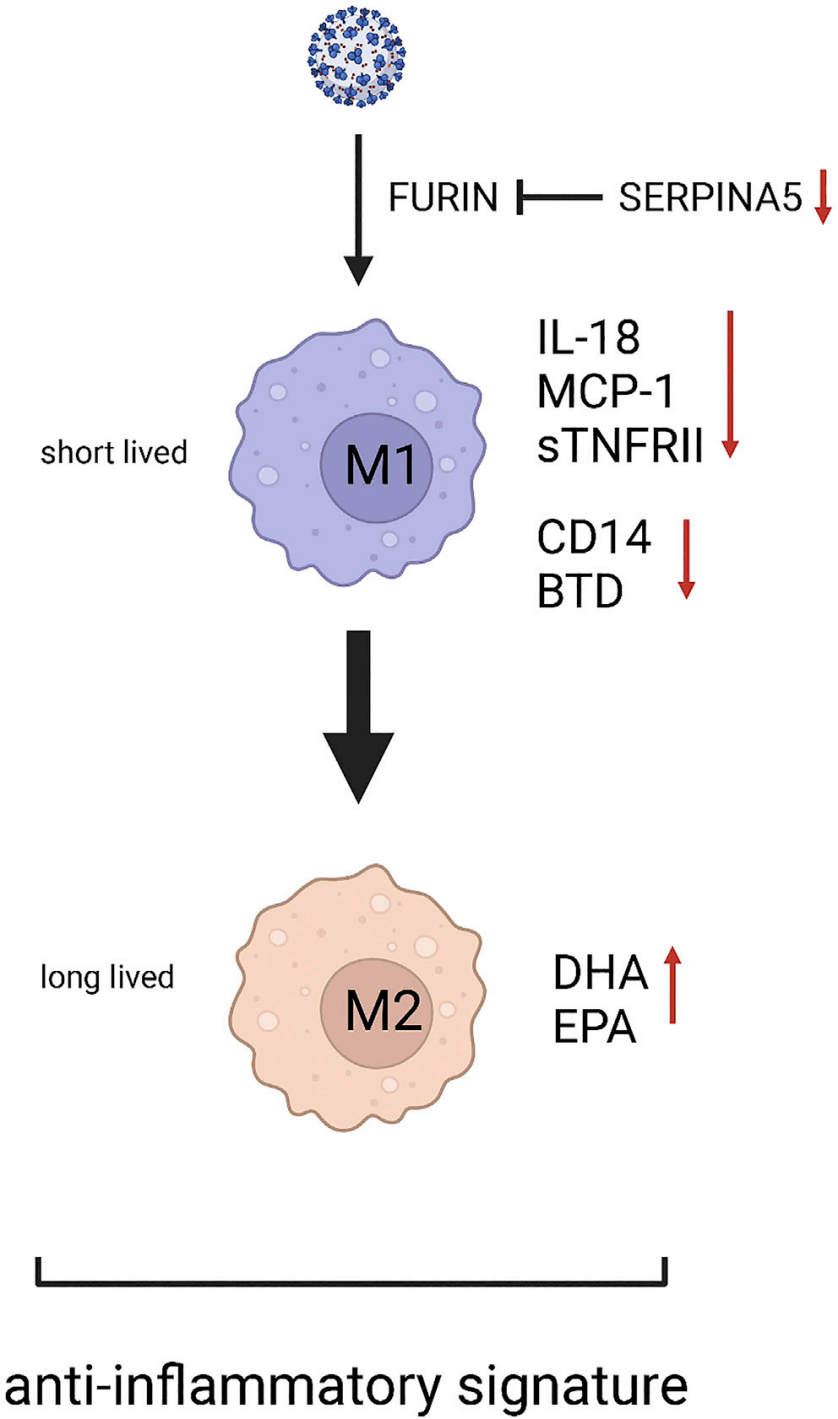
Outline of the suggested pathomechanisms. Monocytes may be specifically affected in patients with LCS In the course of chronic viral infection, there is evidence for a decrease in the occurrence and activities of M1 macrophages accompanied with an increase in M2-like macrophage activities. The indicated M1-derived molecules were found downregulated in patients with LCS, whereas the indicated M2-derived molecules were found up-regulated, indicating an anti-inflammatory signature.

Furthermore, the observed proteome patterns indicate differential monocyte/macrophage polarization and activity between the LCS and the recovered group since the most significantly down-regulated proteins in patients with LCS were found to be derived from macrophages or to directly affect macrophage function (Figure 5). CD14 is a macrophage-specific membrane protein eventually secreted into plasma upon inflammatory activation.^28^ Down-regulation of CD14 may thus indicate less M1-like macrophage responses in patients with LCS. Angiogenin (ANG) is an angiogenic protein also described as anti-bacterial protein secreted by macrophages.^29^ Proteoglycan-4 (PRG4) is mainly expressed by fibroblast-like cells but serves as an important regulator of inflammation^30^ and has been described to act as an essential regulator of synovial macrophage polarization and inflammatory macrophage joint infiltration.^31^ Biotinidase has been described to be essential for basic macrophage functions. Intriguingly, biotinidase deficiency may account for hypotonia, lethargy, cognitive retardation, and seizures^32^ thus potentially linking our observations to the symptom complex of patients with LCS. Finally, Vitamin D binding protein (GC), actually up-regulated in plasma of patients with LCS, may also be involved in macrophage functions, as it is a precursor for the so-called macrophage-activating signal factor.^33^ Furthermore, among the up-regulated proteins in the LCS group, SERPINA1 has been described as characteristic marker of M2-polarized macrophages,^34^ thus additionally giving evidence about alternative macrophage polarization in LCS.

Apart from the proteome analyses, also the observed patterns in the lipidome of patients with LCS provided independent evidence for an anti-inflammatory status. Along those lines, we identified increased levels of DHA, its metabolites and other docosanoids in patients with LCS, which are mainly regarded as anti-inflammatory and may promote tolerogenic macrophage polarization.^35^ Of note, DHA does not only act as anti-inflammatory mediator but high DHA levels are relevant for normal brain function^36^ as well as mitochondrial functions in different cell types.^37^ DHA and other Ω3-fatty acids are preferentially released by calcium-independent phospholipase iPLA2^38^ which is broadly expressed throughout the body and induced upon oxidative stress and mitochondrial damage.^38^ Thus, we hypothesized that the observed increase in Ω3-fatty acids in LCS was a long-term response to a sustained catabolism combined with oxidative stress generated during the COVID-19 infection. In this respect, longitudinal studies addressing these points with more patient are clearly warranted.

An LCS-associated lack of inflammatory processes was also evidenced by the present metabolomics results. The osmolyte taurine, which has been attributed with anti-inflammatory and anti-oxidative properties,^39^ was significantly increased in patients with LCS compared to the other two groups. The metabolite most significantly up-regulated in patients with LCS was hypaphorine (TrpBetaine), an anti-inflammatory alkaloid^21^ described to induce sleep in mice,^22^ which might thus also relate to the chronic fatigue symptoms in LCS. Inversely, hypoxanthine levels, which are among others indicative of tissue hypoxia as found during acute inflammation, were strongly increased in the recovered group, while patients with LCS showed similar levels to the healthy group.

Apart from this overall pattern of anti-inflammation, the metabolome analyses also provided first indications regarding an aberrant amino acid catabolism in LCS. Along those lines, levels of branched chain amino acids were significantly decreased in the LCS group, including both glucogenic as well as ketogenic amino acids. This observation gives evidence for increased energy consumption from protein breakdown, which is in line with reports of increased muscle weakness and sarcopenia due to metabolic alterations after SARS-CoV-2 infection.^40^ Furthermore, tryptophan levels were found down-regulated in the LCS group potentially resulting from prolonged IDO activity, which has also been observed during acute SARS-CoV-2 infection.^40^ Metabolic alterations were also found at the level of triacylglycerols and other complex lipids which were slightly but consistently reduced in the LCS group. Increased levels of fatty acids accompanied by decreased levels of triacylglycerols point to increased activities of endothelial lipase,^41^ which was associated with cognitive impairment.^42^ Intriguingly, this pattern is highly similar to the observations described for CFS,^43^ providing a possible explanation for the fatigue and brain fog symptoms observed in LCS. These findings also underline the potential to introduce novel dietary approaches into tailored rehabilitation regimen. In this regard also strict control of metabolic comorbidities like diabetes, could help in reducing and managing LCS.^44^

From the above observed molecular patterns an overall contribution of alternatively polarized M2 macrophages may be deduced. During acute infection peripheral blood monocytes are strongly affected^45^ and are major contributors to the inflammatory reaction.^46^ While not contributing to viral replication, macrophages may eventually get infected themselves by SARS-CoV-2^47^^,^^48^ and peripheral blood monocytes were described to be significantly altered during COVID-19 infection.^45^ *In vitro* polarized M1-like macrophages express the protease furin (see Figure 4B) which is essential for viral entry into human cells^27^ and might therefore contribute to the course, severity and long-term sequelae of the infection. However, the pro-inflammatory M1 state may be switched, e.g. by oxidized phospholipids accumulated during acute infection, to a tolerogenic M2-like phenotype,^49^^,^^50^ thus becoming long-lived cells coordinating tissue regeneration.^51^ Lipid peroxidation may occur consequent to inflammatory responses also during sterile inflammation such as atherosclerosis^52^ and thus represents a plausible mechanism inducing tolerogenic macrophage polarization after SARS-CoV-2 infection. Polarized tolerogenic macrophages suppress pro-inflammatory cytokines^53^ and induce an anti-inflammatory state,^54^ and are promoted by anti-inflammatory docosanoids,^35^ highly reminiscent to the presently observed molecular pattern characteristic for patients with LCS. As a consequence, here we suggest system-wide alternative macrophage polarization as key cell mechanism accounting for LCS symptoms.

The present data also provide insights into the processes of successful recovery after acute SARS-CoV-2 infection. It is noteworthy that the symptom-free recovered individuals showed alterations compared to the healthy control group in many assessed parameters throughout the different biomolecular compartments. Taken together these findings provide evidence that, even months after acute infection, systemic processes are still active in these individuals. Thus, SARS-CoV-2 infection might leave molecular remnants such as infected macrophages long after symptomatic recovery. Three potential mechanisms have been suggested recently to account for LCS: immune dysregulation, autoimmunity or viral persistence.^55^ Our data are fully compatible with immune dysregulation and viral persistence, but hardly support a general role of autoimmunity, as this should be expected to be associated with a pro-inflammatory pattern.

Thus, the obtained molecular patterns do not only provide first insights into the pathophysiology of COVID-19 sequelae as depicted in Figure 5, but may also provide a first basis for the definition of LCS specific biomarkers. Our broad scale analyses could not detect a unique and specific marker for the disease. However, many significant molecular alterations can be clearly associated with characteristic symptoms of the disease. It is possible to hypothesize regarding the biological causes of these alterations. In addition, it can be envisioned that a combination of presently described proteins (e.g. low SERPINA5), docosanoids (e.g. high DHA) and small metabolites (e.g. high hypaphorine) in patients with characteristic anamnesis and symptoms could help to identify and better define LCS. In this regard, large scale studies to assess the potential sensitivity and specificity of such scores, including the consideration of different SARS-CoV-2 strains, are clearly warranted.

In summary, here we present a distinct multi-omics signature demonstrating a prevalence of anti-inflammatory effector molecules combined with molecular patterns of characteristically altered metabolism detectable in plasma of patients with LCS, offering a unique chance for diagnosis with selected molecular biomarkers and providing novel hypotheses about the pathophysiology of the disease, thus potentially aiding the development of urgently required treatment options.

### Limitations of the study

The disease state “Long Covid Syndrome” is mainly characterized by clinical symptoms and may not only be related to a single causative molecular mechanism. It is to be expected that there will be main aberrations from physiologic pathways associated with the viral infection, that may eventually result in fatigue syndromes including LCS. Molecular profiling provides rich information regarding ongoing processes in human individuals, but may also be distorted by medications or other lifestyle parameters. While this report presents a conclusive pathomechanism potentially accounting for disease symptoms and a potential contributor, i.e. alternatively polarized macrophages, it requires further clinical verification. The study group was of limited size, patients had been infected with only one virus variant, and mainly patients suffering from fatigue syndrome associated with LCS were considered. Thus, the present study cannot claim generality. However, the present data rule out pro-inflammatory mechanisms as general feature of LCS. The focus on a single cell type, here macrophages, is only one important aspect. Other immune cells as well as epithelial cells and endothelial cells will also contribute to the molecular aberrations described in this report and to the characteristic symptoms of LCS. Only ongoing clinical research will show whether the consideration of the specific role of macrophages may help to establish rational therapeutic strategies for patients with LCS.

## STAR★Methods

### Key resources table

**Table:** 

REAGENT or RESOURCE	SOURCE	IDENTIFIER
**Chemicals, peptides, and recombinant proteins**		

ProtiFi S-trap™ micro columns	PROTIFI	N/A
StrataX 33 μm SPE columns	Phenomenex	8B-S100-TAL
Phorbol 12-myristate 13-acetate (PMA)	Sigma-Aldrich	P1585-1MG
Lipopolysaccharides from *Escherichia coli* 055:B5, γ-irradiated, BioXtra, suitable for cell culture	Sigma-Aldrich	L6529-1MG
12S-HETE-d8	Cayman	334570
15S-HETE-d8	Cayman	334230
5-Oxo-ETE-d7	Cayman	334250
11,12-DiHETrE-d11	Cayman	10007975
PGE2-d4	Cayman	314010
20-HETE-d6	Cayman	390030

**Critical commercial assays**		

MxP® Quant 500 Kit (96) - SCIEX	Biocrates life sciences	21094
ProcartaPlex™ Multiplex Immunoassay	Thermo Fisher Scientific	MAN0017980
Elecsys Anti-SARS-CoV-2 S immunoassay	Roche	09 289 267 190
Elecsys Anti-SARS-CoV-2 immunoassay	Roche	09 345 272 190

**Deposited data**		

Plasma proteomics data	ProteomeXchange	PXD036969
Cell lysates of M1 and M2-like macrophages	ProteomeXchange	PXD036972
Supernatants of M1 and M2-like macrophages	ProteomeXchange	PXD036970

**Experimental models: Cell lines**		

U937 cells	ATCC	CLO:0009465

**Software and algorithms**		

MaxQuant 1.6.17.0	max planck institute of biochemistry	www.maxquant.org
Perseus 1.6.14.0	max planck institute of biochemistry	Maxquant.net/perseus/
SwissProt human proteome database version 141219	UniPro consortium	www.uniprot.org
GraphPad Prism 6.07	PraphPad Software	www.graphpad.com
MetIDQ-Oxygen-DB110-3005	Biocrates	N/A

**Other**		

Aurora Series Emitter column 25 cm × 75 μm, 1.6um FSC C18, CSI	ionoptics	AUR2-25075C18A-CSI
Kinetex® 2.6 μm XB-C18 100 Å, LC Column 100 × 2.1 mm, Ea	Phenomenex	00D-4496-AN
MxP Quant 500 kit column system	Biocrates life sciences	21117.1

### Resource availability

#### Lead contact

Further information and requests for resources and reagents should be directed to and will be fulfilled by the lead contact, Christopher Gerner at christopher.gerner@univie.ac.at.

#### Materials availability

This study did not generate new unique reagents.

### Experimental model and subject details

#### Ethics statement

This exploratory study is in compliance with the Helsinki Declaration (Ethical Principles for Medical Research Involving Human Subjects) and was conducted in accordance with the guidelines of research boards at the study site after written informed consent. Recruitment of Long COVID Syndrome patients was approved by the local ethics committee of the Medical University of Vienna under the number EC#1280/2020, while the recruitment of the COVID-19 recovered and the healthy/vaccinated control groups was approved by the local ethics committee of the Medical University of Vienna under the number EC#2262/2020. The recruitment of an independent group including 10 healthy subjects which had not been exposed to SARS-CoV-2 receiving tablets containing 870 mg Omega-3 (420 mg EPA and 330 mg DHA) twice daily in a prospective study design for one week was approved by the local ethics committee of the Medical University of Vienna under the number EC#2250/2020.

#### Patient recruitment and sample acquisition

All study participants were recruited in the timeframe between May and July 2021. LCS patients were recruited at the outpatient ward of the Division of Cardiology of the Department for Internal Medicine II of the Medical University of Vienna, Austria. The age and gender matched recovered/healthy and the healthy/vaccinated study groups were recruited among volunteers after calls at the Vienna General Hospital/Medical University of Vienna and the University of Applied Sciences, Vienna, Austria. All vaccinated participants had received two doses of either the vector-based vaccine Vaxzevria (Astra Zeneca, Oxford, UK) or the mRNA-based vaccine Comirnaty (Pfizer, New York City, NY, USA). Serum and EDTA-anticoagulated plasma were obtained by peripheral venous blood draw. Serum samples were left for 15 min to allow for clotting before centrifugation for 15 min at 1500 g at 4 °C while EDTA plasma samples were immediately centrifuged after blood collection. Following these steps, all samples were immediately frozen at −80 °C until analyses.

Regarding the fourth group of 10 healthy subjects which had not been exposed to SARS-CoV-2, tablets containing 870 mg Omega-3 (420 mg EPA and 330 mg DHA) were administered twice daily in a prospective study design for one week. EDTA-anticoagulated plasma was obtained before start of intake and after intake of the last dose. EDTA plasma samples were immediately centrifuged after blood collection and frozen at −80 °C until analyses.

#### Determination of anti SARS-CoV-2 antibody status

Antibody levels against SARS-CoV-2 Spike protein (anti-S) and Nucleocapsid Protein (anti-N) were determined from sera of the study participants using the Elecsys Anti-SARS-CoV-2 S immunoassay as well as the qualitative Elecsys Anti-SARS-CoV-2 immunoassay (detecting SARS-CoV-2 N protein) on a cobas e801 analyzer (Roche Diagnostics, Rotkreuz, Switzerland). Analyses were performed at the diagnostic laboratory at the Department of Laboratory Medicine, Medical University of Vienna (certified acc. to ISO 9001:2015 and accredited acc. to ISO 15189:2012).

#### Determination of routine laboratory parameters

Total protein, albumin, ferritin, CRP, HDL, LDL, LP(a) were determined using standard routine diagnostic tests on a cobas e801 analyzer (Roche) at the diagnostic laboratory at the Department of Laboratory Medicine, Medical University of Vienna.

#### Determination of serum cytokines

Undiluted blood serum samples were analyzed using the ProcartaPlex™ Multiplex Immunoassay (Human immune monitoring 65 Plex, Thermo Fisher Scientific, Reference Number MAN0017980) according to manufacturer’s instruction. Of the 65 analytes, 51 were below the lower limit of quantification in >95% of all samples (irrespective of group) and were therefore excluded from further analysis. Measurements and analysis of all Human ProcartaPlex Immunoassays were performed on a Luminex 200 instrument (Luminex Corp., Austin, Tx, USA) as described in detail before.^2^

#### Plasma proteomics

For untargeted plasma proteomics analyses, EDTA-anticoagulated plasma samples were diluted 1:20 in lysis buffer (8 M urea, 50 mM TEAB, 5% SDS), heated at 95 °C for 5 min prior the protein concentration was determined using a BCA assay. For enzymatic protein digestion, 20 μg of protein was used and the ProtiFi S-trap technology^56^ applied. Briefly, solubilized protein was reduced and carbamidomethylated by adding 64 mM dithiothreitol (DTT) and 48 mM iodoacetamide (IAA), respectively. Prior to sample loading onto the S-trap mini cartridges, trapping buffer (90% v/v methanol, 0.1M triethylammonium bicarbonate) was added. Thereafter, samples were thoroughly washed and subsequently digested using Trypsin/Lys-C Mix at 37 °C for two hours. Finally, peptides were eluted, dried and stored at −20 °C until LC-MS analyses.

Reconstitution of dried peptide samples was achieved by adding 5 μL of 30% formic acid (FA) containing 4 synthetic standard peptides and subsequent dilution with 40 μL of loading solvent (97.9% H_2_O, 2% ACN, 0.05% trifluoroacetic acid). Thereof, 1 μL were injected into the Dionex Ultimate 3000 nano high performance liquid chromatography (HPLC)-system (Thermo Fisher Scientific), one injection per sample. In order to pre-concentrate peptides prior to chromatographic separation, a pre-column (2 cm × 75 μm C18 Pepmap100; Thermo Fisher Scientific) run at a flow rate of 10 μL/min using mobile phase A (99.9% H_2_O, 0.1% FA) was used. The subsequent peptide separation was achieved on an analytical column (25 cm × 75 μm 1.6 μm C18 Aurora Series emitter column (Ionopticks)) by applying a flow rate of 300 nL/min and using a gradient of 7% to 40% mobile phase B (79.9% ACN, 20% H_2_O, 0.1% FA) over 43 min, resulting in a total LC run time of 85 min including washing and equilibration steps. Mass spectrometric analyses were performed using the timsTOF Pro mass spectrometer (Bruker) equipped with a captive spray ion source run at 1650 V. Further, the timsTOF Pro mass spectrometer was operated in the Parallel Accumulation-Serial Fragmentation (PASEF) mode and a moderate MS data reduction was applied. A scan range (m/z) from 100 to 1700 to record MS and MS/MS spectra and a 1/k0 scan range from 0.60 to 1.60 V.s/cm2 resulting in a ramp time of 100 ms to achieve trapped ion mobility separation were set as further parameters. All experiments were performed with 10 PASEF MS/MS scans per cycle leading to a total cycle time of 1.16 s. Furthermore, the collision energy was ramped as a function of increasing ion mobility from 20 to 59 eV and the quadrupole isolation width was set to 2 Th for m/z < 700 and 3 Th for m/z > 700.

Subsequent LC-MS data analysis was performed using the publicly available software package MaxQuant 1.6.17.0 running the Andromeda search engine.^57^ Protein identification as well as label-free quantification (LFQ) was achieved by searching the raw data against the SwissProt database “homo sapiens” (version 141219 with 20380 entries). General search parameter included an allowed peptide tolerance of 20 ppm, a maximum of 2 missed cleavages, carbamidomethylation on cysteines as fixed modification as well as methionine oxidation and N-terminal protein acetylation as variable modification. A minimum of one unique peptide per protein was set as search criterium for positive identifications. In addition, the “match between runs” option was applied, using a 0.7 min match time window and a match ion mobility window of 0.05 as well as a 20 min alignment time window and an alignment ion mobility of 1. FDR calculation was based on the use of a reversed decoy database, an FDR≤0.01 was set for all peptide and protein identifications.

Further LC-MS data processing and evaluation was accomplished using the Perseus software (version 1.6.14.0).^58^ First, identified proteins were filtered for reversed sequences as well as common contaminants and annotated according to the different study groups. Prior to statistical analysis, LFQ intensity values were transformed (log2(x)), and proteins were additionally filtered for their number of independent identifications (a minimum of 5 identifications in at least one group). Afterwards, missing values were replaced from a normal distribution (width: 0,3; down shift: 1,8).

#### Plasma lipidomics

Frozen EDTA-anticoagulated plasma was freshly thawed on ice. For precipitation of proteins, plasma (300 μL) was mixed with cold EtOH (1.2 mL, abs. 99%, −20°C; AustroAlco) including an internal standard mixture of 12S-HETE-d8, 15S-HETE-d8, 5-Oxo-ETE-d7, 11,12-DiHETrE-d11, PGE2-d4 and 20-HETE-d6 (each 100 nM; Cayman Europe, Tallinn, Estonia). The samples were stored over-night at −20°C. After centrifugation (30 min, 4536 g, 4°C), the supernatant was transferred into a new 15 mL Falcon^TM^ tube. EtOH was evaporated *via* vacuum centrifugation at 37°C until the original sample volume (300 μL) was restored. For solid phase extraction (SPE) samples were loaded onto preconditioned StrataX SPE columns (30 mg mL−1; Phenomenex, Torrance, CA, USA) using Pasteur pipettes. After sample loading, the SPE columns were washed with 5 mL of MS grade water and eluted with ice-cold MeOH (500 μL; MeOH abs.; VWR International, Vienna, Austria) containing 2% formic acid (FA; Sigma-Aldrich). MeOH was evaporated using a gentle nitrogen stream at room temperature and the dried samples were reconstituted in 150 μL reconstitution buffer (H_2_O:ACN:MeOH + 0.2% FA–vol% 65:31.5:3.5). The samples were then transferred into an autosampler held at stored at 4°C and subsequently measured via LC-MS/MS.

For LC-MS analyses, analytes were separated using a Thermo Scientific^TM^ Vanquish^TM^ (UHPLC) system equipped with a Kinetex® C18-column (2.6 μm C18 100 Å, LC Column 150 × 2.1 mm; Phenomenex®) applying a gradient flow profile (mobile phase A: H_2_O + 0.2% FA, mobile phase B: ACN:MeOH (vol% 90:10) + 0.2% FA) starting at 35% B and increasing to 90% B (1–10 min), further increasing to 99% B within 0.5 min and held for 5 min. Solvent B was then decreased to the initial level of 35% within 0.5 min and the column was equilibrated for 4 min, resulting in a total run time of 20 min. The flow rate was kept at 200 μL min−1 and the column oven temperature at 40°C. The injection volume was 20 μL and all samples were analysed in technical duplicates. The Vanquish UHPLC system was coupled to a Q Exactive^TM^ HF Quadrupole-Orbitrap^TM^ high-resolution mass spectrometer (Thermo Fisher Scientific, Austria), equipped with a HESI source for negative ionization to perform the mass spectrometric analysis. The MS scan range was 250-700 m/z with a resolution of 60,000 (at m/z 200) on the MS1 level. A Top 2 method was applied for fragmentation (HCD 24 normalized collision energy), preferable 33 m/z values specific for well-known eicosanoids and precursor molecules from an inclusion list. The resulting fragments were analysed on the MS2 level at a resolution of 15,000 (at m/z 200). Operating in negative ionization mode, a spray voltage of 3.5 kV and a capillary temperature of 253 °C were applied. Sheath gas was set to 46 and the auxiliary gas to 10 (arbitrary units).

For subsequent data analysis, raw files generated by the Q Exactive^TM^ HF Quadrupole-Orbitrap^TM^ high-resolution mass spectrometer were checked manually via Thermo Xcalibur^TM^ 4.1.31.9 (Qual browser) and compared with reference spectra from the Lipid Maps depository library from July 2018.^59^ Peak integration was performed using the TraceFinder^TM^ software package (version 4.1—Thermo Scientific, Vienna, Austria). Principal Component Analysis and volcano plots were generated using the Perseus software (version 1.6.14.0) applying an FDR of 0.05 ^58^. Therefore, data were normalised to the internal standards and missing values were replaced by a constant which corresponds to the half of the lowest normalized area under the curve (nAUC) value of each individual eicosanoid. The ratio of Ω-3/Ω-6 fatty acids depicted in Figure 2 reflects the ratio of the omega-3 polyunsaturated fatty acids eicosapentaenoic acid (EPA) and docosahexaenoic (DHA) to the omega-6 polyunsaturated fatty acids arachidonic acid (AA) and dihomo-gamma-linolenic acid (DGLA).

#### Plasma metabolomics

EDTA plasma samples (10 μL) of study participants were analyzed by a targeted metabolomic assay. Targeted metabolomics experiments were conducted using the MxP® Quant 500 Kit (Biocrates Life Sciences AG, Innsbruck, Austria). We detected 630 analytes, including 40 acylcarnithines, 1 alkaloid, 1 amine oxide, 50 amino acid related metabolite, 14 bile acids, 9 biogenic amines, 7 carboxylic acids, 28 ceramides, 22 cholesteryl esters, 1 cresol, 44 diacylglycerols, 8 dihydroceramides, 12 fatty acids, 90 glycerophospholipids, 34 glycosylceramides, 4 hormones, 4 indole derivatives, 2 nucleobase related metabolites, 15 sphingolipids, 242 triacylglycerols, the sum of hexoses and 1 vitamin/cofactor. A total of 474 metabolites showed signal intensities within the quantification window and were further evaluated. Measurements were carried out using LC-MS and flow injection (FIA)-MS analyses on a Sciex 6500+ series mass spectrometer coupled to an ExionLC AD chromatography system (AB Sciex, Framingham, MA, USA), using the Biocrates MxP Quant 500 kit column system and utilizing the Analyst 1.7.1 software with hotfix 1 (also AB SCIEX). All required standards, quality controls and eluents were included in the kit, as well as the chromatographic column for the LC-MS/MS analysis part. Phenyl isothiocyanate (Sigma-Aldrich, St. Louis, USA) was purchased separately and was used for derivatization of amino acids and biogenic amines according to the kit manual. Preparation of the measurement worklist as well as data validation and evaluation were performed with the software supplied with the kit (MetIDQ-Oxygen-DB110-3005, Biocrates Life Sciences). The heatmap including 126 triacylglycerols (Table S4) was generated by dividing the concentration of each lipid through the average concentration of this lipid over all samples using Excel.

#### Cell culture and differentiation of U937 cells

U937 cell line was cultured in RPMI medium (1X with L-Glutamine; Gibco, Thermo Fischer Scientific, Austria) supplemented with 1% Penicillin/Streptomycin (Sigma-Aldrich, Austria) and 10% Fetal Calf Serum (FCS, Sigma-Aldrich, Austria) in T25 polystyrene cell culture flasks for suspension cells (Sarstedt, Austria) at 37 °C and 5% CO_2_. Cells were counted using a MOXI Z Mini Automated Cell Counter (ORFLO Technologies, USA) using Moxi Z Type M Cassettes (ORFLO Technologies, USA). Based on this, 2 × 10^6^ cells were used for each differentiation approach and seeded in T25 polystyrene cell culture flasks with cell growth surface for adherent cells (Sarstedt, Austria).

Differentiation of U937 cells into M1-like macrophages was induced by adding 100 ng/mL Phorbol 12-myristate 13-acetate (PMA, ≥99%, Sigma-Aldrich, Austria) (d0). After 48 h (d2), the cell culture medium was exchanged and fresh full medium supplemented with 100 ng/mL LPS (Lipopolysaccharides from *Escherichia coli* 055:B5, γ-irradiated, BioXtra, Sigma-Aldrich, Austria) was added. Again, after 48 h (d4), M1-like macrophages were washed twice with PBS and further incubated with 3 mL of serum free RPMI for 4 h. Thereafter, supernatants were harvested, precipitated using 12 mL cold EtOH (abs. 99%, −20°C; AustroAlco) including an internal standard mixture of 12S-HETE-d8, 15S-HETE-d8, 5-Oxo-ETE-d7, 11,12-DiHETrE-d11, PGE2-d4 and 20-HETE-d6 (each 100 nM; Cayman Europe, Tallinn, Estonia) and stored at −20 °C until further processing. M1-like macrophages were lysed in 200 μL of a 4% SDC buffer containing 100 mM Tris-HCl (pH 8.5), immediately heat-treated at 95°C for 5 minutes, ultra-sonicated and stored at −20 °C until further processing.

M2-like macrophage differentiation of U937 cells was achieved by first adding 100 ng/mL PMA (d0) for 24 h to the full medium. Afterwards, 50 ng/mL M-CSF (ImmunoTools, Friesoythe, Germany) were directly added to the culture medium (d1) for a total of 72 h before the medium was exchanged and cells cultivated in fresh full medium supplemented again with 50 ng/mL M-CSF (d4) for another 72 h. After this (d6), cells were incubated in fresh medium containing 20 ng/mL IL-4 (ImmunoTools) for 24 h to induce the M2-like phenotype. At day 7 (d7) of the differentiation process, M2-like macrophages were washed twice with PBS and further incubated with 3 mL of serum free RPMI for 4 h. Sample harvesting was performed as described above. M1-like macrophage differentiation as well as M2-like macrophage differentiation of U937 cells were carried out in triplicates.

#### Sample preparation and LC-MS analyses of M1-like and M2-like macrophages

Precipitated FCS-free cell supernatants were centrifuged (30 min, 4536 g, 4°C) and the supernatant was transferred into new 15 mL Falcon^TM^ tubes for lipid extraction as described above. The protein pellet corresponding to the secreted proteins was dissolved in 4% SDC buffer containing 100 mM Tris-HCl (pH 8.5), ultra-sonicated and heat-treated at 95°C for 5 minutes. Protein concentration of supernatants as well as cell lysates was determined using a BCA assay. For proteomic analyses, an adapted version of the EasyPhos workflow was applied.^60^ In short, 20 μg of protein was reduced and alkylated in one step using 100 mM TCEP and 400 mM 2-CAM, respectively. Subsequent enzymatic digestion was performed with a Trypsin/Lys-C mixture (1:100 Enzyme to Substrate ratio) at 37 °C for 18 h. For desalting, peptide solution was first dried to approximately 20 μL, mixed with loading buffer containing 1% TFA in isopropanol and loaded on SDB-RPS StageTips. After washing twice, peptides were eluted with 60% ACN and 0.005% ammonium hydroxide solution, dried and stored at −20 °C until LC-MS analyses.

LC-MS analyses of the supernatants of M1-like and M2-like macrophages were carried out as describe above (plasma proteomics and plasma lipidomics) with slight adaptions regarding the proteomics analyses. All samples were measured in technical duplicates and biological triplicates, resulting in 6 measurements per sample type. In case of secretome analysis 5 μL of resuspended sample were injected into the Dionex Ultimate 3000 nano high performance liquid chromatography (HPLC)-system (Thermo Fisher Scientific) but using the same gradient as for plasma proteomics. Regarding the proteomic analyses of cell lysates, again 5 μL of resuspended sample were injected but an adapted LC-gradient from 8% to 40% mobile phase B over 90 min, resulting in a total LC run time of 135 min including washing and equilibration steps, was applied.

### Quantification and statistical analysis

With regard to serum cytokine analysis, values from the three groups (pg/mL) were compared by one-way ANOVA followed by Tukey’s multiple comparisons test.

With regard to proteome, lipidome and metabolome profiling experiments, Principal Component Analyses, two-sided t-tests as well as statistics for volcano plots were performed using the Perseus software (version 1.6.14.0) and applying an FDR (permutation-based with 250 permutations) of 0.05 and a S0 of 0.1, whereby S0 controls the relative importance of *t*-test p-value and difference between the means. Histograms were generated using GraphPad Prism Version 6.07 (2015).

## Data Availability

The proteome analysis datasets presented in this study can be found in online repositories. The names of the repository/repositories and accession number(s) can be found below: http://www.proteomexchange.org/, TBA, ProteomeXchange, identifier PXD036969 (plasma proteins), PXD036972 (macrophage cell lysates) and PXD036970 (macrophage supernatants). This paper does not report original code. Any additional information required to reanalyze the data reported in this paper is available from the lead contact upon request.

## Supplemental information

Document S1. Figures S1 and S2

Table S1. Student's t-test statistics between long COVID-19 and recovered, long COVID-19 and healthy as well as recovered and healthy are shown, related to Figure 1 For each identified protein, log2 label-free quantification (LFQ) intensities, numbers of identified peptides, numbers of identified unique peptides as well as the sequence coverage are listed.

Table S2. Student's t-test statistics between long COVID-19 and recovered, long COVID-19 and healthy as well as recovered and healthy are shown, related to Figure 2 For each identified eicosanoid normalized area under the curve (nAUC) values are listed.

Table S3. Student's t-test statistics between long COVID-19 and recovered, long COVID-19 and healthy as well as recovered and healthy are shown, related to Figure 3 For each identified metabolite log2 concentrations in μM are listed.

Table S4. Heatmap data matrix, related to Figure 3

## References

[1] de la Rica R., Borges M., Gonzalez-Freire M. (2020). COVID-19: in the eye of the cytokine storm. Front. Immunol..

[2] Kovarik J.J., Kämpf A.K., Gasser F., Herdina A.N., Breuer M., Kaltenecker C.C., Wahrmann M., Haindl S., Mayer F., Traby L. (2021). Identification of immune activation markers in the early onset of COVID-19 infection. Front. Cell. Infect. Microbiol..

[3] Reddy K., Rogers A.J., McAuley D.F. (2020). Delving beneath the surface of hyperinflammation in COVID-19. Lancet. Rheumatol..

[4] Rubin R. (2020). As their numbers grow, COVID-19 "long haulers" stump experts. JAMA.

[5] Yong S.J. (2021). Long COVID or post-COVID-19 syndrome: putative pathophysiology, risk factors, and treatments. Infect. Dis..

[6] Blomberg B., Mohn K.G.I., Brokstad K.A., Zhou F., Linchausen D.W., Hansen B.A., Lartey S., Onyango T.B., Kuwelker K., Sævik M. (2021). Long COVID in a prospective cohort of home-isolated patients. Nat. Med..

[7] Shah R., Ali F.M., Nixon S.J., Ingram J.R., Salek S.M., Finlay A.Y. (2021). Measuring the impact of COVID-19 on the quality of life of the survivors, partners and family members: a cross-sectional international online survey. BMJ Open.

[8] Ahmed H., Patel K., Greenwood D.C., Halpin S., Lewthwaite P., Salawu A., Eyre L., Breen A., O'Connor R., Jones A., Sivan M. (2020). Long-term clinical outcomes in survivors of severe acute respiratory syndrome and Middle East respiratory syndrome coronavirus outbreaks after hospitalisation or ICU admission: a systematic review and meta-analysis. J. Rehabil. Med..

[9] Cervia C., Zurbuchen Y., Taeschler P., Ballouz T., Menges D., Hasler S., Adamo S., Raeber M.E., Bächli E., Rudiger A. (2022). Immunoglobulin signature predicts risk of post-acute COVID-19 syndrome. Nat. Commun..

[10] Sacks D., Baxter B., Campbell B.C.V., Carpenter J.S., Cognard C., Dippel D., Eesa M., Fischer U., Hausegger K., Hirsch J.A., From the American Association of Neurological Surgeons, American Society of Neuroradiology, Cardiovascular and Interventional Radiology Society of Europe, Canadian Interventional Radiology Association, Congress of Neurological Surgeons, European Society of Minimally Invasive Neurological Therapy, European Society of Neuroradiology, European Stroke Organization, Society for Cardiovascular Angiography and Interventions, Society of Interventional Radiology, Society of NeuroInterventional Surgery, World Stroke Organization (2018). Multisociety consensus quality improvement revised consensus statement for endovascular therapy of acute ischemic stroke. Int. J. Stroke.

[11] Su Y., Yuan D., Chen D.G., Ng R.H., Wang K., Choi J., Li S., Hong S., Zhang R., Xie J. (2022). Multiple early factors anticipate post-acute COVID-19 sequelae. Cell.

[12] Mehandru S., Merad M. (2022). Pathological sequelae of long-haul COVID. Nat. Immunol..

[13] Muqaku B., Pils D., Mader J.C., Aust S., Mangold A., Muqaku L., Slany A., Del Favero G., Gerner C. (2020). Neutrophil extracellular trap formation correlates with favorable overall survival in high grade ovarian cancer. Cancers.

[14] Reichl B., Niederstaetter L., Boegl T., Neuditschko B., Bileck A., Gojo J., Buchberger W., Peyrl A., Gerner C. (2020). Determination of a tumor-promoting microenvironment in recurrent medulloblastoma: a multi-omics study of cerebrospinal fluid. Cancers.

[15] Muqaku B., Eisinger M., Meier S.M., Tahir A., Pukrop T., Haferkamp S., Slany A., Reichle A., Gerner C. (2017). Multi-omics analysis of serum samples demonstrates reprogramming of organ functions via systemic calcium mobilization and platelet activation in metastatic melanoma. Mol. Cell. Proteomics.

[16] Franzke B., Bileck A., Unterberger S., Aschauer R., Zöhrer P.A., Draxler A., Strasser E.M., Wessner B., Gerner C., Wagner K.H. (2022). The plasma proteome is favorably modified by a high protein diet but not by additional resistance training in older adults: a 17-week randomized controlled trial. Front. Nutr..

[17] Dennis E.A., Norris P.C. (2015). Eicosanoid storm in infection and inflammation. Nat. Rev. Immunol..

[18] Leitner G.C., Hagn G., Niederstaetter L., Bileck A., Plessl-Walder K., Horvath M., Kolovratova V., Tanzmann A., Tolios A., Rabitsch W. (2022). INTERCEPT pathogen reduction in platelet concentrates, in contrast to gamma irradiation, induces the formation of trans-arachidonic acids and affects eicosanoid release during storage. Biomolecules.

[19] Altmann R., Hausmann M., Spöttl T., Gruber M., Bull A.W., Menzel K., Vogl D., Herfarth H., Schölmerich J., Falk W., Rogler G. (2007). 13-Oxo-ODE is an endogenous ligand for PPARgamma in human colonic epithelial cells. Biochem. Pharmacol..

[20] Germain A., Ruppert D., Levine S.M., Hanson M.R. (2017). Metabolic profiling of a myalgic encephalomyelitis/chronic fatigue syndrome discovery cohort reveals disturbances in fatty acid and lipid metabolism. Mol. Biosyst..

[21] Sun H., Zhu X., Cai W., Qiu L. (2017). Hypaphorine attenuates lipopolysaccharide-induced endothelial inflammation via regulation of TLR4 and PPAR-gamma dependent on PI3K/Akt/mTOR signal pathway. Int. J. Mol. Sci..

[22] Ozawa M., Honda K., Nakai I., Kishida A., Ohsaki A. (2008). Hypaphorine, an indole alkaloid from Erythrina velutina, induced sleep on normal mice. Bioorg. Med. Chem. Lett..

[23] Atri C., Guerfali F.Z., Laouini D. (2018). Role of human macrophage polarization in inflammation during infectious diseases. Int. J. Mol. Sci..

[24] Cardoso A.P., Pinto M.L., Castro F., Costa Â.M., Marques-Magalhães Â., Canha-Borges A., Cruz T., Velho S., Oliveira M.J. (2021). The immunosuppressive and pro-tumor functions of CCL18 at the tumor microenvironment. Cytokine Growth Factor Rev..

[25] Kwon M.Y., Park E., Lee S.J., Chung S.W. (2015). Heme oxygenase-1 accelerates erastin-induced ferroptotic cell death. Oncotarget.

[26] Toro A., Ruiz M.S., Lage-Vickers S., Sanchis P., Sabater A., Pascual G., Seniuk R., Cascardo F., Ledesma-Bazan S., Vilicich F. (2022). A journey into the clinical relevance of heme oxygenase 1 for human inflammatory disease and viral clearance: why does it matter on the COVID-19 scene?. Antioxidants.

[27] Bestle D., Heindl M.R., Limburg H., Van Lam van T., Pilgram O., Moulton H., Stein D.A., Hardes K., Eickmann M., Dolnik O. (2020). TMPRSS2 and furin are both essential for proteolytic activation of SARS-CoV-2 in human airway cells. Life Sci. Alliance.

[28] Hiki N., Berger D., Prigl C., Boelke E., Wiedeck H., Seidelmann M., Staib L., Kaminishi M., Oohara T., Beger H.G. (1998). Endotoxin binding and elimination by monocytes: secretion of soluble CD14 represents an inducible mechanism counteracting reduced expression of membrane CD14 in patients with sepsis and in a patient with paroxysmal nocturnal hemoglobinuria. Infect. Immun..

[29] Noschka R., Gerbl F., Löffler F., Kubis J., Rodríguez A.A., Mayer D., Grieshober M., Holch A., Raasholm M., Forssmann W.G. (2020). Unbiased identification of angiogenin as an endogenous antimicrobial protein with activity against virulent Mycobacterium tuberculosis. Front. Microbiol..

[30] Das N., Schmidt T.A., Krawetz R.J., Dufour A. (2019). Proteoglycan 4: from mere lubricant to regulator of tissue homeostasis and inflammation: does proteoglycan 4 have the ability to buffer the inflammatory response?. Bioessays.

[31] Qadri M., Jay G.D., Zhang L.X., Schmidt T.A., Totonchy J., Elsaid K.A. (2021). Proteoglycan-4 is an essential regulator of synovial macrophage polarization and inflammatory macrophage joint infiltration. Arthritis Res. Ther..

[32] Akgun A., Sen A., Onal H. (2021). Clinical, biochemical and genotypical characteristics in biotinidase deficiency. J. Pediatr. Endocrinol. Metab..

[33] Yamamoto N., Homma S. (1991). Vitamin D3 binding protein (group-specific component) is a precursor for the macrophage-activating signal factor from lysophosphatidylcholine-treated lymphocytes. Proc. Natl. Acad. Sci. USA.

[34] Lurier E.B., Dalton D., Dampier W., Raman P., Nassiri S., Ferraro N.M., Rajagopalan R., Sarmady M., Spiller K.L. (2017). Transcriptome analysis of IL-10-stimulated (M2c) macrophages by next-generation sequencing. Immunobiology.

[35] Caron J.P., Gandy J.C., Brown J.L., Sordillo L.M. (2018). Docosahexaenoic acid-derived oxidized lipid metabolites modulate the inflammatory response of lipolysaccharide-stimulated macrophages. Prostaglandins Other Lipid Mediat..

[36] Dyall S.C. (2015). Long-chain omega-3 fatty acids and the brain: a review of the independent and shared effects of EPA, DPA and DHA. Front. Aging Neurosci..

[37] Stanley W.C., Khairallah R.J., Dabkowski E.R. (2012). Update on lipids and mitochondrial function: impact of dietary n-3 polyunsaturated fatty acids. Curr. Opin. Clin. Nutr. Metab. Care.

[38] Sun G.Y., Geng X., Teng T., Yang B., Appenteng M.K., Greenlief C.M., Lee J.C. (2021). Dynamic role of phospholipases A2 in health and diseases in the central nervous system. Cells.

[39] Marcinkiewicz J., Kontny E. (2014). Taurine and inflammatory diseases. Amino Acids.

[40] Piotrowicz K., Gąsowski J., Michel J.P., Veronese N. (2021). Post-COVID-19 acute sarcopenia: physiopathology and management. Aging Clin. Exp. Res..

[41] Khetarpal S.A., Vitali C., Levin M.G., Klarin D., Park J., Pampana A., Millar J.S., Kuwano T., Sugasini D., Subbaiah P.V. (2021). Endothelial lipase mediates efficient lipolysis of triglyceride-rich lipoproteins. PLoS Genet..

[42] Yun S.M., Park J.Y., Seo S.W., Song J. (2019). Association of plasma endothelial lipase levels on cognitive impairment. BMC Psychiatr..

[43] Naviaux R.K., Naviaux J.C., Li K., Bright A.T., Alaynick W.A., Wang L., Baxter A., Nathan N., Anderson W., Gordon E. (2016). Metabolic features of chronic fatigue syndrome. Proc. Natl. Acad. Sci. USA.

[44] Raveendran A.V., Misra A. (2021). Post COVID-19 syndrome ("Long COVID") and diabetes: challenges in diagnosis and management. Diabetes Metab. Syndr..

[45] Siska P.J., Decking S.M., Babl N., Matos C., Bruss C., Singer K., Klitzke J., Schön M., Simeth J., Köstler J. (2021). Metabolic imbalance of T cells in COVID-19 is hallmarked by basigin and mitigated by dexamethasone. J. Clin. Invest..

[46] Kim Y.M., Shin E.C. (2021). Type I and III interferon responses in SARS-CoV-2 infection. Exp. Mol. Med..

[47] Hui K.P.Y., Cheung M.C., Perera R.A.P.M., Ng K.C., Bui C.H.T., Ho J.C.W., Ng M.M.T., Kuok D.I.T., Shih K.C., Tsao S.W. (2020). Tropism, replication competence, and innate immune responses of the coronavirus SARS-CoV-2 in human respiratory tract and conjunctiva: an analysis in ex-vivo and in-vitro cultures. Lancet Respir. Med..

[48] Grant R.A., Morales-Nebreda L., Markov N.S., Swaminathan S., Querrey M., Guzman E.R., Abbott D.A., Donnelly H.K., Donayre A., Goldberg I.A. (2021). Circuits between infected macrophages and T cells in SARS-CoV-2 pneumonia. Nature.

[49] Zhivaki D., Kagan J.C. (2022). Innate immune detection of lipid oxidation as a threat assessment strategy. Nat. Rev. Immunol..

[50] Gundacker N.C., Haudek V.J., Wimmer H., Slany A., Griss J., Bochkov V., Zielinski C., Wagner O., Stöckl J., Gerner C. (2009). Cytoplasmic proteome and secretome profiles of differently stimulated human dendritic cells. J. Proteome Res..

[51] Suuring M., Moreau A. (2021). Regulatory macrophages and tolerogenic dendritic cells in myeloid regulatory cell-based therapies. Int. J. Mol. Sci..

[52] Zhong S., Li L., Shen X., Li Q., Xu W., Wang X., Tao Y., Yin H. (2019). An update on lipid oxidation and inflammation in cardiovascular diseases. Free Radic. Biol. Med..

[53] Shrivastava R., Shukla N. (2019). Attributes of alternatively activated (M2) macrophages. Life Sci..

[54] Naito Y., Takagi T., Higashimura Y. (2014). Heme oxygenase-1 and anti-inflammatory M2 macrophages. Arch. Biochem. Biophys..

[55] Brodin P., Casari G., Townsend L., O'Farrelly C., Tancevski I., Löffler-Ragg J., Mogensen T.H., Casanova J.L., COVID Human Genetic Effort (2022). Studying severe long COVID to understand post-infectious disorders beyond COVID-19. Nat. Med..

[56] Zougman A., Selby P.J., Banks R.E. (2014). Suspension trapping (STrap) sample preparation method for bottom-up proteomics analysis. Proteomics.

[57] Cox J., Mann M. (2008). MaxQuant enables high peptide identification rates, individualized p.p.b.-range mass accuracies and proteome-wide protein quantification. Nat. Biotechnol..

[58] Cox J., Mann M. (2012). 1D and 2D annotation enrichment: a statistical method integrating quantitative proteomics with complementary high-throughput data. BMC Bioinf..

[59] Fahy E., Sud M., Cotter D., Subramaniam S. (2007). LIPID MAPS online tools for lipid research. Nucleic Acids Res..

[60] Humphrey S.J., Karayel O., James D.E., Mann M. (2018). High-throughput and high-sensitivity phosphoproteomics with the EasyPhos platform. Nat. Protoc..

