# Supplementary material for: A multi-omics based anti-inflammatory immune signature characterizes long COVID-19 syndrome
Source: iScience. 2022 Dec 5;26(1):105717. doi: 10.1016/j.isci.2022.105717 (PMC9719844; doi:10.1016/j.isci.2022.105717)
Supplement: Document S1. Figures S1 and S2 [file mmc1.pdf]

iScience, Volume *26*

Supplemental information

A multi-omics based anti-inflammatory immune signature characterizes long COVID-19 syndrome

Johannes J. Kovarik, Andrea Bileck, Gerhard Hagn, Samuel M. Meier-Menches, Tobias Frey, Anna Kaempf, Marlene Hollenstein, Tarik Shoumariyeh, Lukas Skos, Birgit Reiter, Marlene C. Gerner, Andreas Spannbauer, Ena Hasimbegovic, Doreen Schmidl, Gerhard Garhöfer, Mariann Gyöngyösi, Klaus G. Schmetterer, and Christopher Gerner

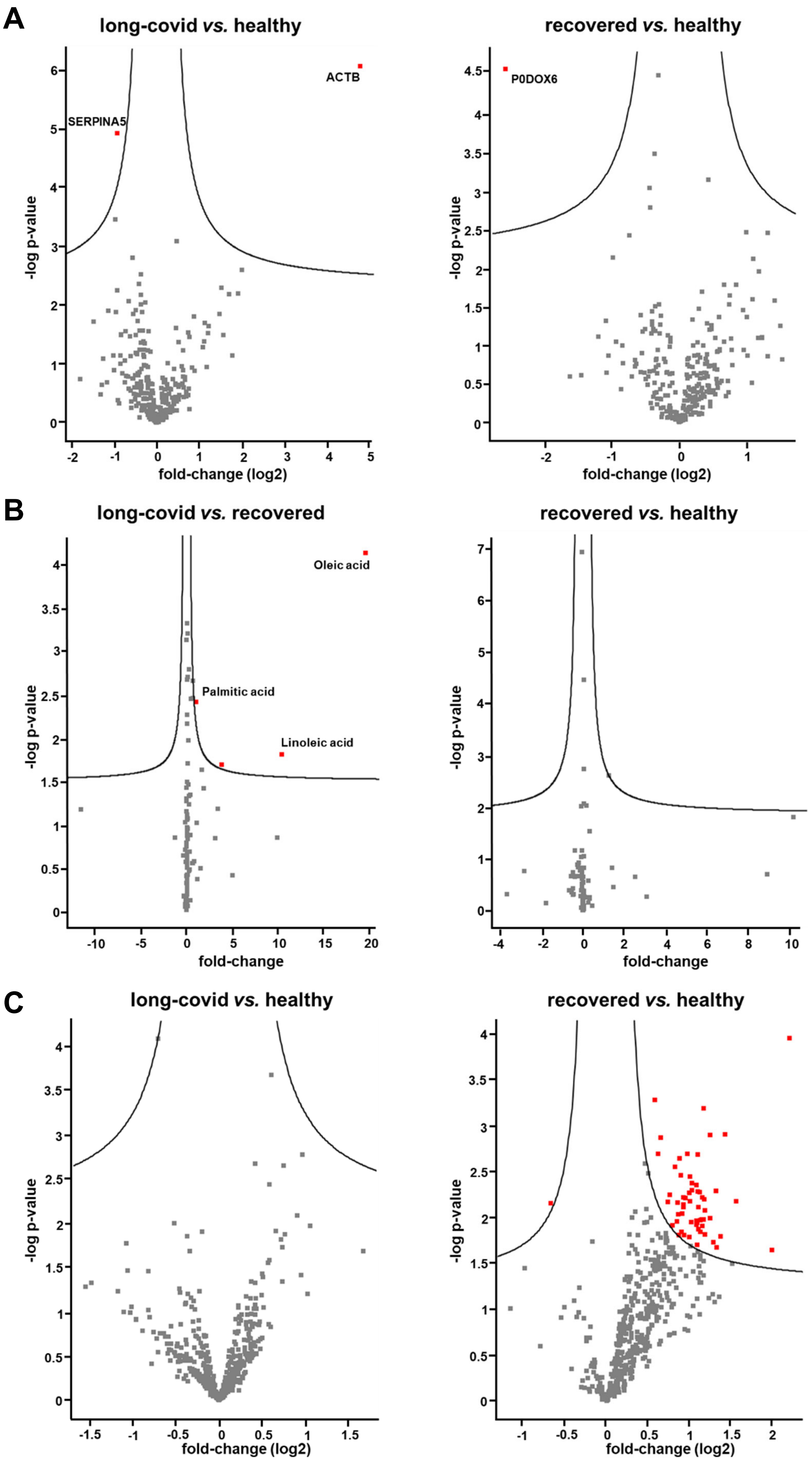

Supplementary Figure S1 related to Figures 1, 2 and 3: Volcano plots

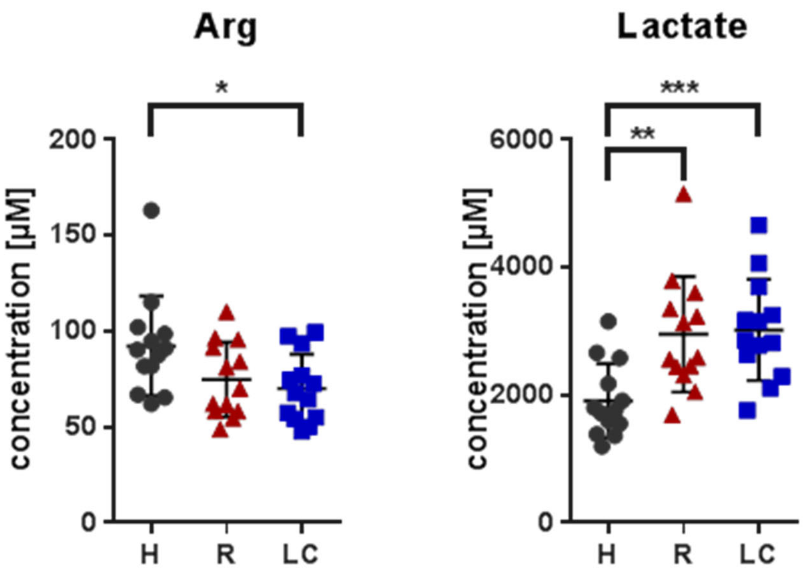

Supplementary Figure S2 related to Figure 3: Lactate concentration in Plasma of healthy controls (H), recovered (R) and long COVID (LC) patients.
